# TC-N19, a novel dual inhibitor of EGFR and cMET, efficiently overcomes EGFR-TKI resistance in non-small-cell lung cancer cells

**DOI:** 10.1038/cddis.2016.192

**Published:** 2016-06-30

**Authors:** D-W Wu, T-C Chen, H-S Huang, H Lee

**Affiliations:** 1 Graduate Institute of Cancer Biology and Drug Discovery, College of Medical Science and Technology, Taipei Medical University, Taipei, Taiwan

## Abstract

Epidermal growth factor receptor-tyrosine kinase inhibitors (EGFR-TKIs) show a clinical benefit when used to treat patients with EGFR-mutated non-small-cell lung cancer (NSCLC), but this treatment unfortunately fails in patients with TKI-resistant tumors. We here provide evidence that TC-N19 (N19), a novel dual inhibitor of EGFR and cMET, efficiently overcomes the EGFR-TKI resistance in EGFR-mutated NSCLC cells via simultaneous degradation of both proteins by ubiquitin proteasomes. Comparison with HSP90 inhibitor treatment and knockdown of EGFR and cMET by small hairpin RNAs reveal that the reduction of EGFR and cMET expression by N19 is responsible for overcoming the intrinsic TKI resistance mediated by paxillin (PXN) in high PXN-expressing cells, PXN-overexpressing PC9 cells (PC9-PXN), the EGFR-T790M-mediated TKI resistance in H1975 and CL97 cells, and the acquired resistance to gefitinib in gefitinib-resistant PC9 cells (PC9GR). Annexin V-PI staining assay showed that the induction of apoptosis in NSCLC cells by N19 depended on the reduction in levels of both proteins. Xenograft tumor formation in nude mice induced by a PC9-PXN-stable clone and by PC9GR cells was nearly completely suppressed by N19 treatment, with no changes in animal body weight. MTT assays of normal lung cells and reticulocytes showed no cytotoxicity responses to N19. In summary, N19 may act as a novel dual inhibitor of EGFR and cMET that induces apoptosis in TKI-resistant EGFR-mutated NSCLC cells and suppresses xenograft tumor formation. We suggest that N19 may be a potential new-generation TKI or HSP90 inhibitor used for treatment of NSCLC patients who show resistance to current TKI-targeting therapies.

Mutations in the epidermal growth factor receptor (EGFR) are recognized as promising biomarkers for therapies using tyrosine kinase inhibitors (TKIs) as treatments for non-small-cell lung cancer (NSCLC).^1, 2, 3^ Resistance to TKIs frequently occurs in EGFR-mutated NSCLC patients who have undergone TKI treatment and this resistance is considered to represent an acquired (secondary) resistance.^4, 5^

The mechanisms of intrinsic (primary) TKI resistance are not fully understood, but paxillin (PXN) overexpression confers intrinsic TKI resistance in NSCLC via modulation of Mcl-1 and BIM protein stability due to ERK activation.^6^ The combination of TKI with the ERK inhibitor selumetinib is reported to improve TKI sensitivity and outcomes in cell and animal models.^7, 8^ Unfortunately, no benefit has yet been established for combining an ERK inhibitor and a TKI as a treatment for NSCLC patients.

The most common acquired resistance mutation in the EGFR is T790M at exon 20.^9, 10^ The EGFR-T790M mutation and cMET amplification account for 50–60% and 5–20%, respectively, of the observed EGFR-TKI resistance in NSCLC patients.^9, 10^ The protein expression and phosphorylation of EGFR-T790M and cMET have been associated with both intrinsic and acquired resistance to TKI-targeting therapy in these patients. Therefore, the development of a new generation of EGFR-TKI and cMET inhibitors represents a critical strategy for overcoming EGFR-TKI resistance in NSCLC.^11, 12, 13, 14, 15, 16, 17, 18, 19^ Unfortunately, EGFR-independent mechanisms of acquired resistance to AZD9291, a third-generation TKI, have already been reported in EGFR-E790M-positive NSCLC patients.^20^

Mouse lung cancer models that express the EGFR mutations Del19-T790M or L858R-T790M, each with concurrent cMET overexpression, showed no significant tumor regression in response to monotherapy that targeted EGFR or cMET alone.^21^ By contrast, combination therapies that simultaneously targeted EGFR and cMET were highly efficacious against EGFR-TKI-resistant tumors codriven by Del19-T790M or L858R-T790M and cMET. Despite this promising result, however, the same combined approach of EGFR-TKI+cMET inhibitors failed when used in clinical trials involving human patients with EGFR-mutated NSCLC.^22^ This setback has prompted the search for a dual inhibitor that could target both EGFR and cMET simultaneously, as this might show greater effectiveness than the combination of TKI+cMET inhibitors against EGFR-TKI-resistant NSCLC.

A new anthraquinone derivative, the small-molecule TC-19 (N19), has received a US patent as an inhibitor of cell proliferation in NSCLC cells (NSC777201) and it has also shown effective inhibition of cell growth in DU-145 and PC-3 cell lines.^23^ In this study, we provide new evidence that N19 may act as a dual inhibitor of both EGFR and cMET against PXN-mediated EGFR-TKI resistance in NSCLC cells and that it acts by promoting the degradation of both proteins by ubiquitin proteasomes.

## Results

### N19 is more effective than gefitinib at inducing apoptotic inhibition of cell viability and colony formation in EGFR-mutated NSCLC cells

**Figure 1:**
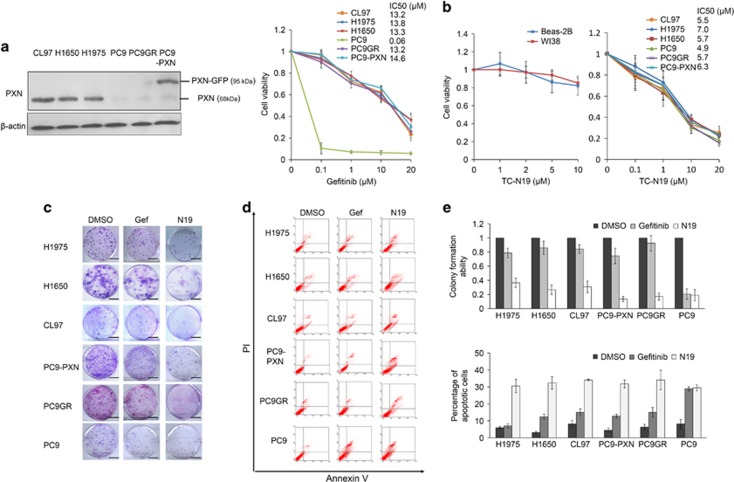
N19 treatment inhibits cell growth and induces apoptosis on EGFR-mutated NSCLC cells. (**a**) EGFR-mutated lung cancer cells (H1975, H1650, CL97, PC9-PXN, PC9GR, and PC9) were treated with gefitinib at the indicated concentrations for 48 h and cell viability and IC_50_ (half-maximal inhibitory concentration) was analyzed by the MTT (3-(4,5-dimethythiazol-2-yl)-2,5-diphenyl tetrazolium bromide) assay. Error bars are shown as mean±S.D.; *n*=3. (**b**) Normal lung cells (WI38 and Beas-2B) and EGFR-mutated lung cancer cells (H1975, H1650, CL97, PC9-PXN, PC9GR, and PC9) were treated with N19 at the indicated concentrations for 48 h and cell viability and IC_50_ was analyzed by the MTT assay. (**c** and **d**) Colony formation (**c**) and annexinV-PI staining assay (**d**) were performed in six EGFR-mutated lung cancer cell types subjected to gefitinib or N19 treatment. (**e**) The relative colony formation ability and percentage of apoptotic cells modulated by gefitinib or N19 were summarized in these six cell types. All data were collected from three independent experiments. The mean value and S.D. were indicated as the column with error bars

PXN confers intrinsic TKI resistance in EGFR-mutated NSCLC cells.^6^ The IC_50_ value for gefitinib in six EGFR-mutated NSCLC cell lines was evaluated by the MTT assay. The IC_50_ value for gefitinib in H1975, H1650, CL97 and PC9GR (gefitinib-resistant PC9 cells) cells ranged from 13.2 to 13.8 *μ*M. The lowest IC_50_ value was observed in PC9 cells, but the IC_50_ value was markedly increased (to 14.6 *μ*M) by ectopic PXN expression in those cells (Figure 1a). Interestingly, the IC_50_ value for N19 in these cell lines ranged from 5.5 to 7.0 *μ*M, but a relatively lower IC_50_ value (4.9 *μ*M) was observed for N19 in PC9 cells. N19 showed no cytotoxicity in normal WI38 and Beas-2B lung cell lines, as determined by MTT assays (Figure 1b).

Representative colony growth on agar plates is shown in Figure 1c. N19 was more effective than gefitinib at inhibiting colony formation in all cell types, except for the PC9 cells (Figure 1e, upper panel). Representative apoptotic profiles of these cells following gefitinib or N19 treatment and Annexin V-PI staining analysis are shown in Figure 1d. The percentage of apoptotic cells was higher following N19 treatment than following gefitinib treatment in all cell types, except the PC9 cells (Figure 1e, lower panel). These results clearly indicated that greater inhibition of cell viability and colony formation was obtained with N19 than with gefitinib in EGFR-mutated NSCLC cells and that this inhibition occurred via apoptosis.

### N19 kills EGFR-mutated NSCLC cells via apoptosis triggered by degradation of EGFR and cMET proteins

**Figure 2:**
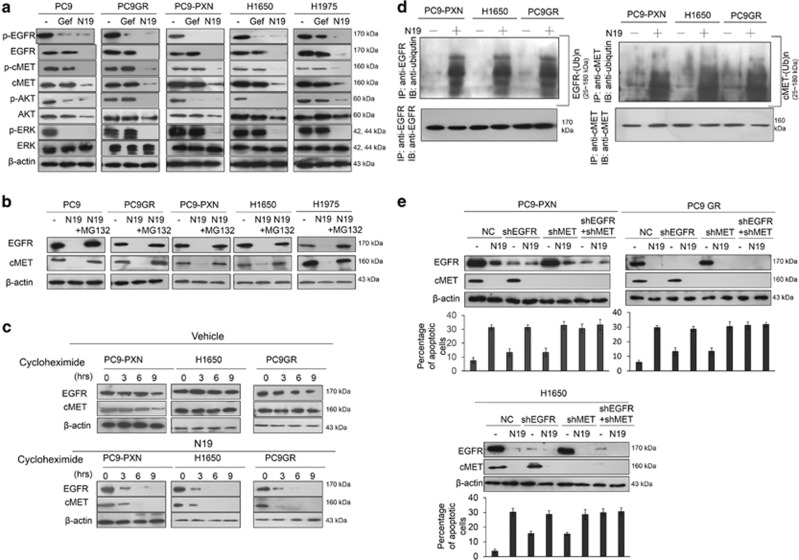
N19 promotes ubiquitination to suppress the expression of EGFR and cMET proteins and their downstream signaling. (**a**) The effect of N19 on EGFR and cMET signaling. At 48 h after gefitinib (10 *μ*M) or N19 (10 *μ*M) treatment, the cell lysates were harvested and analyzed for the signaling alteration by western blot with indicated antibodies. (**b**) Inhibition of the proteasome pathway stabilized EGFR and cMET after N19 treatment. Cells were treated with or without MG132, or N19, or both, and analyzed by western blot. (**c**) N19 treatment decreased the half-life of endogenous EGFR and cMET proteins. PC9-PXN, H1650, and PC9GR cells treated with 100 *μ*g/ml of cycloheximide for the indicated hours with or without 10 *μ*M N19. The protein levels of EGFR and cMET were assessed by western blot. (**d**) The effect of N19 treatment on the polyubiquitination of EGFR and cMET. Cells were treated with N19 at 10 *μ*M in PC9-PXN, H1650, and PC9GR cells, respectively, for 5 h. Polyubiquitination of EGFR and cMET were assessed by western blot in the IP. (**e**) The effect of EGFR or/and cMET knockdown on the apoptosis induced by N19 treatment. PC9-PXN, H1650, and PC9GR cells transfected with EGFR or/and cMET shRNAs were treated with 10 *μ*M N19 for 24 h. The cell apoptosis was measured by annexinV-PI staining assay using a flow cytometry. All data were collected from three independent experiments. The mean value and S.D. were indicated as the column with error bars

We examined the possibility that the death of EGFR-mutated NSCLC cells in response to N19 could occur through the degradation of EGFR and cMET proteins. Western blotting indicated significant reductions in the expression levels of p-EGFR and p-AKT following gefitinib treatment of PC9, PXN-overexpressing PC9 (PC9-PXN), and H1650 cells, but not after gefitinib treatment of PC9GR and H1975 cells (Figure 2a). The expression levels of EGFR, AKT, ERK, and cMET were unchanged by gefitinib treatment in all these cell types. Interestingly, the expressions of p-EGFR, EGFR, p-cMET, cMET, p-AKT, AKT, and p-ERK were essentially suppressed by N19 treatment in these five cell types (Figure 2a). Similar changes in these protein expressions were also observed in response to gefitinib or N19 treatment in high PXN-expressing CL97 cells (Supplementary Figure 1). However, the reduction in EGFR and cMET expression by N19 in these cell types was reversed in the presence of MG132, when compared with cells without N19 treatment (Figure 2b).

A pulse-chase experiment using PC9-PXN, H1650, and PC9GR cells indicated that the expression of EGFR and cMET proteins was gradually decreased by N19 over the indicated time intervals; however, the levels of both proteins were relatively unchanged following treatment with vehicle alone (Figure 2c). The ubiquitin ladder of EGFR and cMET proteins was clearly evident in these cell types following N19 treatment, but was absent in the vehicle controls (Figure 2d). Therefore, the degradation of EGFR and cMET proteins by N19 appears to occur through a post-translational mechanism mediated by proteasome ubiquitin, rather than through modulation of the translation machinery.

We next tested whether the cell apoptosis induced by N19 occurred through degradation of EGFR and cMET proteins. Three cell types (PC9-PXN, PC9GR, and H1650) were transfected with a small hairpin RNA (shRNA) of EGFR (shEGFR) and shcMET, singly or in combination, and then treated with N19. Western blotting showed that the expression levels of EGFR and cMET were significantly reduced by N19 treatment in these cell types transfected with nonspecific shRNA control (Figure 2e). The expressions of EGFR and cMET protein were further reduced by in cells transfected with shEGFR, shcMET, or shEGFR+shcMET and subjected to N19 treatment (Figure 2e). The percentage of apoptotic cells varied with the treatments and depended on the expression levels of EGFR and cMET in these cell types (Figure 2e). These results suggest that N19 kills EGFR-mutated NSCLC cells via apoptosis predominantly through degradation of EGFR and cMET proteins by ubiquitin proteasomes.

### N19 likely acts as an HSP90 inhibitor and kills EGFR-mutated NSCLC cells via simultaneous degradation of EGFR and cMET both proteins by ubiquitin proteasomes

**Figure 3:**
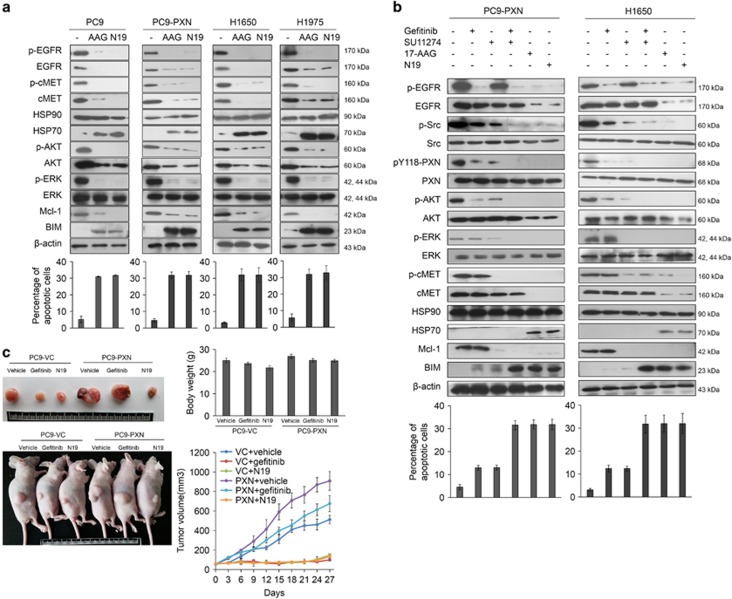
N19 promotes the EGFR and cMET protein degradation via likely acting as HSP90 inhibition. (**a**) The effect of N19 on EGFR and cMET degradation via HSP90 inhibition. At 48 h after 17-AAG (10 *μ*M) or N19 (10 *μ*M) treatment, the cell lysates were harvested and analyzed for the signaling alteration by western blot with indicated antibodies. (**b**) PC9-PXN and H1650 cells were treated with gefitinib, SU11274, gefitinib+SU11274, 17-AAG or N19 for 48 h. The cell apoptosis was measured by annexinV-PI staining assay using a flow cytometry. The cell lysates were harvested and analyzed for the signaling alteration by western blot with indicated antibodies. (**c**) The effect of N19 on tumor growth. The tumor volume was measured as described in Materials and Methods (*n*=5, each group). Photographs show the representative tumor burdens in mice after drug administration for 27 days via intraperitoneal injection. Representative samples of tumor excised on day 27. The effect of N19 on the body weights of mice. Mean±S.D. values were calculated from the tumor volume and body weights of five nude mice in each group

EGFR and cMET are client proteins of HSP90.^24, 25, 26^ We therefore hypothesized that N19 could act as an HSP90 inhibitor to kill EGFR-mutated NSCLC cells via degradation of EGFR and cMET proteins by ubiquitin proteasomes. Western blotting showed that EGFR and cMET expressions in PC9, PC9-PXN, H1650, and H1975 cells were almost completely suppressed by the HSP90 inhibitor 17-AAG or by N19. The expression levels of p-AKT and p-ERK in these cell types were also strongly reduced by 17-AAG or N19 treatment (Figure 3a), whereas the same treatments significantly elevated the expression level of HSP70 in these cell types. The increase in Mcl-1 and decrease in BIM in these cell types were reversed by 17-AAG or N19 treatments. The changes in the levels of Mcl-1 and BIM proteins were consistent with the percentages of apoptotic cells observed after 17-AAG or N19 treatment (Figure 3a, upper and lower panels).

These observations supported the findings of our previous study,^6^ where an increase in Mcl-1 and a decrease in BIM expression were responsible for the EGFR-TKI resistance mediated by PXN overexpression. PC9-PXN and H1650 cells were treated with gefitinib, the cMET inhibitor SU11274, a combination of gefitinib+SU11274, 17-AAG, or N19 to determine whether PXN-mediated TKI resistance could be overcome by gefitinib+SU11274, as well as by 17-AAG or N19. Western blotting indicated that the expression levels of p-EGFR, p-Src, pY118-PXN, p-AKT, p-ERK, and p-cMET were strongly reduced by gefitinib+SU11274, 17-AAG, or N19 in both cell types, but were only partially reduced by gefitinib or SU11274 alone (Figure 3b). The appearance of HSP70 expression was observed in both cell types, but only following 17-AAG or N19 treatment and not the other treatments (Figure 3b). The expressions of Mcl-1 and BIM were reversed by gefitinib+SU11274 and this reversal was also observed following 17-AAG or N19 treatment. Similarly high percentages of apoptotic cells were observed in both cell types following gefitinib+SU11274, 17-AAG, or N19 treatment but not following treatment with gefitinib or SU11274 alone (Figure 3b). Immunoprecipitation (IP) analysis indicated that the interaction of HSP90 with EGFR or cMET in H1650 cells was decreased by N19 treatment in the presence or absence of MG132 (Supplementary Figure 2). These results suggest that N19 likely acts as an HSP90 inhibitor and kills EGFR-mutated NSCLC cells by apoptosis via degradation of EGFR and cMET proteins.

We next compared the suppression of the growth of subcutaneous tumors in nude mice induced by a PC9-PXN-stable clone by N19 and gefitinib treatment. The representative tumor burden and mice with subcutaneous tumors are shown in Figure 3c. The tumor burden in nude mice induced by PC9-VC- or PC9-PXN-stable clone was nearly completely suppressed by N19. A suppressive effect of gefitinib was also observed in the tumor burden of nude mice induced by vector control (VC) cells. However, the tumor burden in nude mice induced by the PC9-PXN-stable clone or PC9-VC cells gradually increased over 27 days when these mice were treated with gefitinib or vehicle. The body weight of the mice was unchanged by N19 or gefitinib treatment during the 27 days study. Similar inhibitory effects were observed for N19 on PC9GR-induced tumor burdens in both cell and animal models (Supplementary Figure 3). These results clearly indicated that N19 completely suppresses tumor growth induced by the PC9-PXN-stable clone and by PC9GR cells. We therefore suggest that N19 efficiently suppresses xenograft tumor formation by cells with both intrinsic and acquired TKI resistance.

## Discussion

These findings provide evidence that N19 may overcome intrinsic TKI resistance mediated by PXN overexpression and destroy EGFR-mutated NSCLC cells via an apoptotic pathway. The acquired TKI resistance mediated by T790M mutations in H1975 and CL97 cells, and in PC9GR cells that express low levels of PXN, was also overcome by N19 (Figure 1b). Therefore, N19 efficiently kills EGFR-mutated NSCLC cells with either intrinsic or acquired TKI resistance by promoting simultaneous degradation of both EGFR and cMET proteins by ubiquitin proteasomes. Our previous report indicated that phosphorylation of PXN at Y118 and Y31 by the Src signaling may confer cisplatin resistance due to upregulation of Bcl-2 transcription.^27^ Accumulating evidence now indicates that EGFR and cMET signaling both have critical roles in the activation of Src signaling.^28, 29, 30, 31^ PXN phosphorylation by Src activation confers cisplatin and TKI resistance in EGFR-wild-type and EGFR-mutated NSCLC cells via ERK activation.^6, 27^ Therefore, the degradation of EGFR and cMET proteins promoted by N19 may overcome EGFR-TKI resistance via inhibition of PXN phosphorylation due to inactivation of Src and ERK signaling. N19 may not only overcome PXN-mediated intrinsic TKI resistance but may also overcome acquired TKI resistance mediated by T790M EGFR mutation (H1975 and CL97) or mediated by acquired gefitinib resistance in PC9GR.

Butein, a natural phytochemical, may act as a dual inhibitor of EGFR and cMET to inhibit cell proliferation in gefitinib-resistant lung cancer cells via inhibition of kinase activities that phosphorylate both proteins.^32^ Several studies have demonstrated that a combined use of EGFR-cMET inhibitors can overcome T790M-EGFR- and cMET-mediated TKI resistance in NSCLC cells, but these resistances cannot be overcome by a cMET inhibitor alone.^17, 21, 33, 34, 35^ The findings in the present study for the EGFR-TKI-resistant cells treated with gefitinib+SU11274 or shEGFR+shMET combinations were consistent with these previous results (Figure 2e).^17, 21, 33, 34, 35^ We have provided evidence that N19 acts as a novel dual inhibitor, promoting the degradation of EGFR and cMET proteins via ubiquitin proteasomes to overcome both intrinsic and acquired TKI resistance in EGFR-mutated NSCLC cells.

HSP90 is a molecular chaperone that is crucial for the stability and function of many proteins essential for cell survival.^36, 37^ Many oncogenes, including tyrosine kinases, transcription factors, and cell-cycle regulatory proteins, are client proteins of HSP90.^38^ Inhibition of HSP90 causes client protein degradations via ubiquitin proteasomes, and this, in turn, suppresses cell survival and tumor growth.^36, 38, 39, 40, 41, 42^ Among these client proteins, AKT, EGFR, and cMET are targeted by HSP90 inhibitors and may have important roles in overcoming EGFR-TKI resistance in NSCLC cells.^43, 44, 45^ HSP90 inhibition overcomes HGF-triggered resistance to TKIs in EGFR-mutated NSCLC cells by decreasing the expressions of the client proteins AKT, EGFR, and cMET. However, no evidence indicates that these client proteins are responsible for EGFR-TKI resistance in NSCLC cells.^43^ A phase I/II clinical trial of the HSP90 inhibitors rataspimycin (IPI-504), ganetespib (STA-9090), and NVP-AUY922 (VER52296) conducted in NSCLC patients has revealed only a partial clinical benefit in EGFR-mutated NSCLC patients.^46, 47, 48^ Unfortunately, these HSP90 inhibitors caused liver dysfunction and night blindness, which halted the clinical trials.^46, 48, 49^ We examined the possibility that N19 could cause degradation of other HSP90 client proteins, including BRAF, HIF-1*α*, IGF1R, and MYC. Western blotting showed that the degradation of BRAF and HIF-1*α* proteins was relatively lower following N19 treatment than following 17-AAG treatment at the same concentration (Supplementary Figure 4). Molecular docking analysis indicated that the affinity of N19 binding to HSP90 was similar with an HSP90 inhibitor ganetespib binding to HSP90 (Supplementary Figure 5). We therefore suggest that N19 appears to act as an HSP90 inhibitor and promotes the degradation of some, but not all, HSP90 client proteins.

We used the MTT assay to determine whether N19 could induce cytotoxicity in retinal pigment epithelial cells (ARPE-19). We observed no cytotoxicity in response to N19 treatment of ARPE-19 cells in concentration ranges up to 10 *μ*M. However, a significant cytotoxicity was observed in ARPE-19 cells subjected to 17-AAG or 17-DMAG treatment (Supplementary Figure 6). Therefore, N19 likely acts as an HSP90 inhibitor and may overcome EGFR-TKI resistance and destroy EGFR-mutated NSCLC cells via degradation of EGFR and cMET proteins by ubiquitin proteasomes. The lack of cytotoxicity of N19 in ARPE-19 reticular cells supports this notion. We therefore suggest that N19 might represent a new generation of EGFR-TKI or HSP90 inhibitor that act as novel dual inhibitors of EGFR and cMET and efficiently overcome intrinsic and acquired EGFR-TKI resistance in NSCLC cells.

## Materials and Methods

### Chemicals and antibodies

Gefitinib, SU11274, 17-DMAG, and 17-AAG were obtained from Selleckchem (Houston, TX, USA). All other chemicals were acquired from Sigma Chemical (St. Louis, MO, USA), unless otherwise indicated. Anti-EGFR, anti-total ERK, anti-phosphorylated (p)-ERK, anti-total AKT, and anti-p-AKT antibodies were obtained from Cell Signaling (Danvers, MA, USA). Anti-HSP70 and anti-HSP90 antibodies were obtained from Genetex (Irvine, CA, USA). All other antibodies were purchased from Santa Cruz Biotechnology (Dallas, TX, USA).

### Cell lines

PC9, PC9GR, and CL97 cells were kindly provided by Drs. P-C Yang and James C-H Yang (Department of Internal Medicine and Graduate Institute of Oncology, College of Medicine, National Taiwan University, Taiwan). The other lung cancer cell lines were obtained from the American Type Culture Collection and cultured as described previously. The EGFR mutation patterns of the cell lines used in this study were shown in Supplementary Table 1.

### *In vivo* animal model experiments

The tumor cells were injected subcutaneously into the backs of 4–5-week-old female Balb/c nude mice. The xenograft tumor size was measured every 3 days and the tumor volume was determined as (lengthxwidth^2^)/2. When tumors grew to 50 mm^3^, mice were randomized to the following groups: vehicle (DMSO), gefitinib (5 mg/kg), or N19 (5 mg/kg). Drugs were administered by intraperitoneal injection every 3 days.

### Plasmid constructs and transfection

The PXN overexpression plasmid was kindly provided by Dr Salgia (The University of Chicago, Chicago, IL, USA). EGFR (TRCN0000121068) and cMET (TRCN0000009850) were purchased from National RNAi Core Facility, Academia Sinica (Taipei City, Taiwan). These plasmids were transiently transfected into lung cancer cells (1 × 10^6^) using the Turbofect reagent (Formentas, Glen Burnie, MD, USA). After 48 h, cells were harvested to assay in subsequent experiments.

### MTT cytotoxicity assay

Before treatment, the cells in the exponential growth phase were pretreated with overexpression and knockdown plasmids for 24 h. The *in vitro* cytotoxic effects of these treatments were determined by MTT assay (at 550 nm) and the cell viability was expressed as a percentage of the control (untreated) cells (% of control).

### Annexin V-PI staining analysis

The cells were collected by trypsinization and centrifugation at 1000 × *g* for 5 min. Following resuspension in binding buffer (10 mM HEPES-NaOH, 140 mM NaCl, 2.5 mM CaCl_2_) at a final cell density of 1–2 × 10^6^ cells per ml, 100 *μ*l of a single-cell suspension (1–2 × 10^5^ cells) was incubated with 5 *μ*l Annexin V-FITC and 5 *μ*l PI for 15 min at room temperature in the dark. After addition of 400 *μ*l of binding buffer, the samples were analyzed with a BD FACS Calibur flow cytometer (BD Biosciences, San Jose, CA, USA) within 1 h. For each sample, 10 000 events were counted.

### Colony formation assay

Cells were plated in six-well plates in complement media overnight. After incubation, the culture media were replaced by fresh medium containing gefitinib, N19, and DMSO as vehicle control for 48 h. These treated cells were cultured in the newly medium supplemented with 10% FBS for another 10 days. Before the pictures of these colonies were taken, cells were stained with 0.01% crystal violet for 1 h at room temperature.

### Western blotting and IP

Western blotting and IP was performed as described previously.^50^ In brief, total cell lysates were prepared in immunoprecipitated lysis buffer (20 mM Tris, pH 7.5, 100 mM sodium chloride, 1% IGEPAL CA-630, 100 *μ*M Na_3_VO_4_, 50 mM NaF, 30 mM sodium pyrophosphate) containing complete protease inhibitor cocktail with or without EDTA (Roche Diagnostics, Basel, Switzerland). Protein concentrations were measured by the Bio-Rad Protein Assay (Bio-Rad, Richmond, CA, USA). For IP, proteins were immunoprecipitated by 1 *μ*g of primary antibodies and protein A beads (Sigma, Danvers, MA, USA) for 4 h and then washed three times per sample with the lysis buffer. Protein samples were separated by 10% sodium dodecyl sulfate-polyacrylamide gels, transferred onto polyvinylidene difluoride membranes (Millipore, Billerica, MA, USA), and finally immunoblotted with primary antibodies. Primer antibodies for immunoblotting were used at 1 : 500 to 1 : 1000 dilution. Horseradish peroxidase-conjugated anti-mouse, goat, and rabbit secondary antibodies (Santa Cruz Biotechnology, Dallas, TX, USA) were performed at 1 : 5000 dilution. Protein signals were detected by chemiluminescent reagents (Amersham Pharmacia, Piscataway, NJ, USA).

## Abbreviations

EGFR: epidermal growth factor receptor
TKI: tyrosine kinase inhibitor
NSCLC: non-small-cell lung cancer
PXN: paxillin; N19, TC-19
PC9GR: gefitinib-resistant PC9 cell
PC9-PXN: PXN-overexpressing PC9
shRNA: small hairpin RNA
VC: vector control
