# Supplementary material for: TC-N19, a novel dual inhibitor of EGFR and cMET, efficiently overcomes EGFR-TKI resistance in non-small-cell lung cancer cells
Source: Cell Death Dis. 2016 Jun 30;7(6):e2290–. doi: 10.1038/cddis.2016.192 (PMC5108342; doi:10.1038/cddis.2016.192)
Supplement: Supplementary Information [file cddis2016192x1.doc]

**TC-N19, a novel dual inhibitor of EGFR and cMET, efficiently overcomes EGFR-TKI resistance in non-small cell lung cancer cells**

De-Wei Wu, Tsung-Chih Chen, Hsu-Shan Huang, and Huei Lee

Graduate Institute of Cancer Biology and Drug Discovery, College of Medical Science and Technology, Taipei Medical University, Taipei, Taiwan.

**Supplementary Figure 1. N19 suppress the expression of EGFR and cMET proteins and their downstream signaling in CL97 cells.** The effect of N19 on EGFR and cMET signaling. Forty-eight hours after gefitinib (10 μM) or N19 (10 μM) treatment, the cell lysates were harvested and analyzed for the signaling alteration by western blot with indicated antibodies.

**Supplementary Figure 2. The interaction of HSP90 with EGFR or cMET in H1650 cells evaluated by immunoprecipitation (IP) analysis was decreased by N19 treatment in the presence or absence of MG132**. H1650 cells were treated with 10 μM N19 for 43 hrs and then the cells were treated with MG132 for additional 5 hrs. The cells lysates were immunoprecipitated with anti-HSP90- conjugated beads. Immunoprecipitates were evaluated EGFR and cMET expression by western blotting. β-Actin was used as a protein loading control.

**Supplementary Figure 3. N19 promotes the EGFR and cMET protein degradation in PC9GR cells via likely as HSP90 inhibition.** (a) The effect of N19 on EGFR and cMET degradation via HSP90 inhibition. Forty-eight hours after 17-AAG (10 μM) or N19 (10 μM) treatment, the cell lysates were harvested and analyzed for the signaling alteration by western blot with indicated antibodies. (b) PC9GR cells were treated with gefitinib, SU11274, gefitinib plus SU11274, 17-AAG or N19 for 48 hrs. The cell apoptosis was measured by annexinV-PI staining assay using a flow cytometry. The cell lysates were harvested and analyzed for the signaling alteration by western blot with indicated antibodies. All data were collected from three independent experiments. The mean value and standard deviation were indicated as the column with error bars. (c) The effect of N19 on tumor growth. The tumor volume was measured as described in Materials and Methods (n=5, each group). Photographs show the representative tumor burdens in mice after drug administration for 27 days via intraperitoneal injection. Representative samples of tumor excised on day 27. The effect of N19 on the body weights of mice. Mean ± SD values were calculated from the tumor volume and body weights of five nude mice in each group.

**Supplementary Figure 4. N19 treatment decreased the client protein of HSP90.** H1650 cells were treated with a dose-dependent manner of N19 or 17-AAG for 48 hrs. The cell lysates were harvested and analyzed for the client protein alterations by western blot with indicated antibodies.

**Supplementary Figure 5.** N19 show higher specificity for docking to HSP90, likely as Ganetespib. The docking value and structure for N19 docking to HSP90.

**Supplementary Figure 6. No cytotoxicity in response to N19 treatment of ARPE-19 cells**. MTT and annexinV-PI staining assay were performed in ARPE cells subjected to N19, 17-AAG or 17-DMAG treatment. All data were collected from three independent experiments. The mean value and standard deviation were indicated as the column with error bars.

**Supplementary Figure 1**

**Supplementary Figure 2**


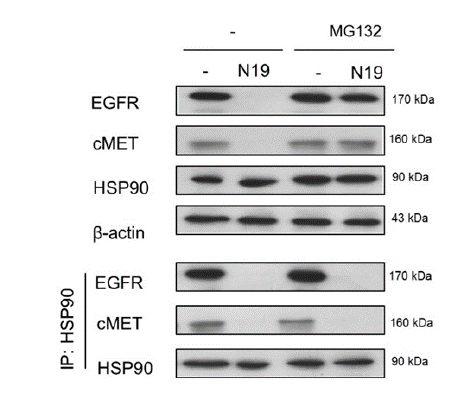


**Supplementary Figure 3**


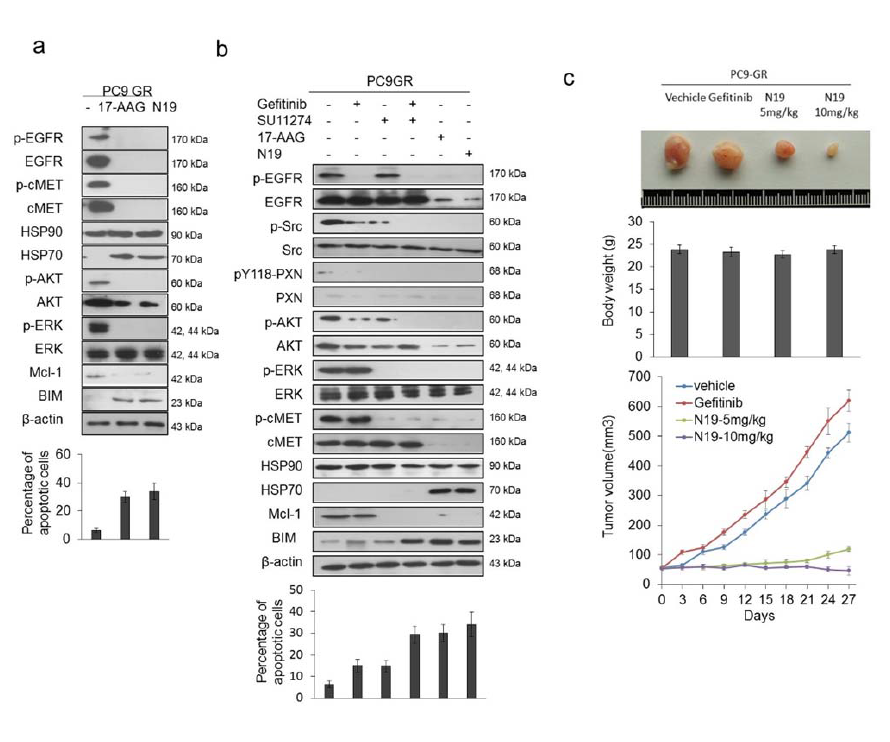


**Supplementary Figure 4**


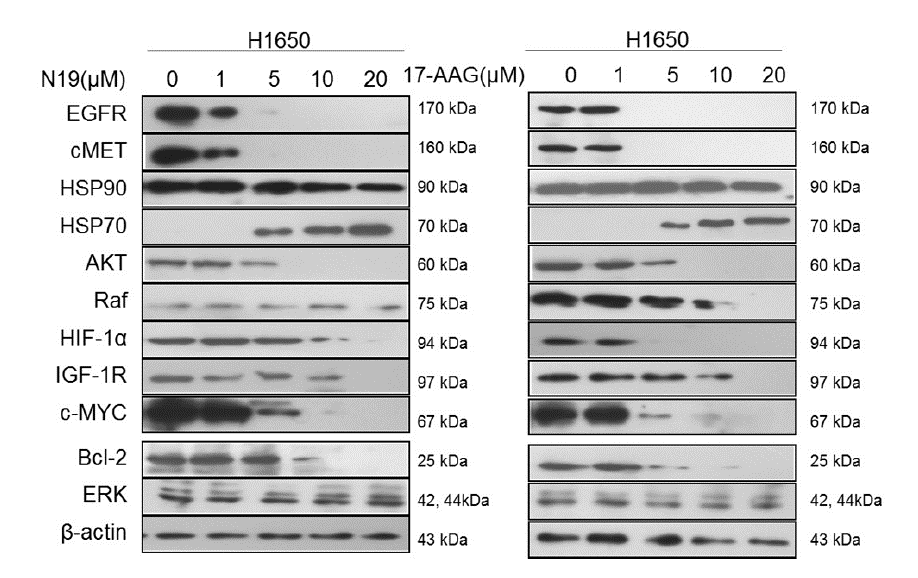


**Supplementary Figure 5**

**
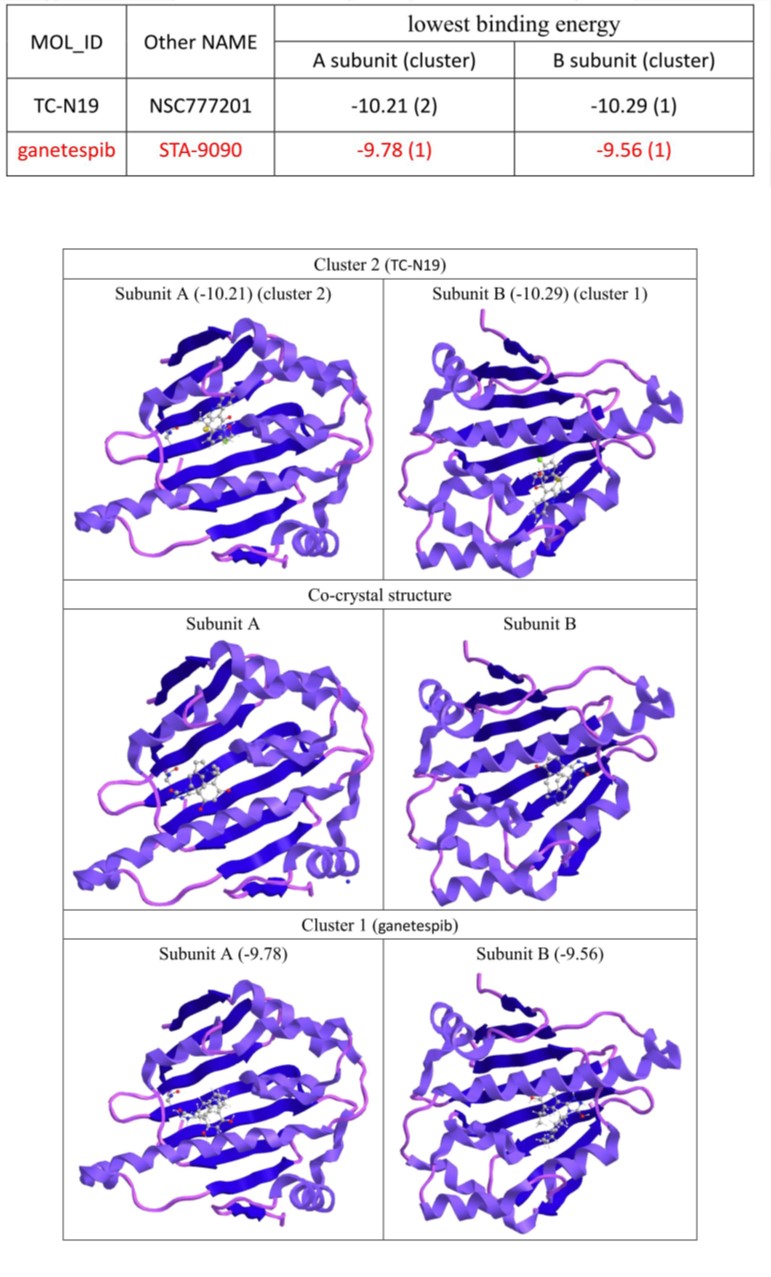
**

**Supplementary Figure 6**


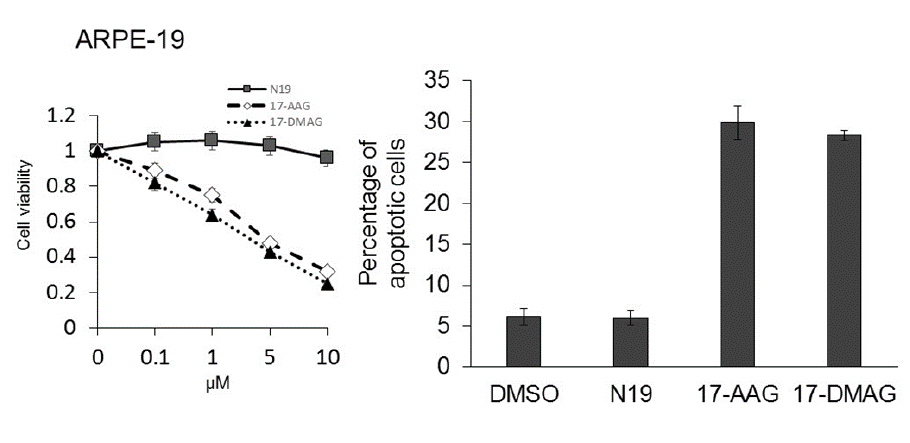


**Supplementary Table 1. EGFR mutation status in lung adenocarcinoma used in this study.**

|  | **EGFR Mutation** |
| --- | --- |
| **CL97** | G719A/T790M |
| **H1650** | delE746-A750 |
| **H1975** | L858R/T790M |
| **PC9** | delE746-A750 |
| **PC9GR** | delE746-A750 |
| **PC9-PXN** | delE746-A750 |
